# A Spinal Cavernous Malformation in Multiple Cerebral Cavernous Venous Malformations Syndrome

**DOI:** 10.5334/jbsr.2986

**Published:** 2023-01-02

**Authors:** Guillaume Vangrinsven, Phyllis Vanwalleghem, Özkan Özsarlak

**Affiliations:** 1 University Hospital Antwerp, BE; 2 Monica Hospitals, BE

**Keywords:** spinal cavernous malformations, familial multiple cavernous malformation syndrome, spinal hemorrhage, MRI

## Abstract

**Teaching Point:** In patients with familial multiple cavernous malformation syndrome with acute focal neurological deficit, a symptomatic spinal cavernous malformation must be included in the differential diagnosis.

## Case History

**Figure 1:**
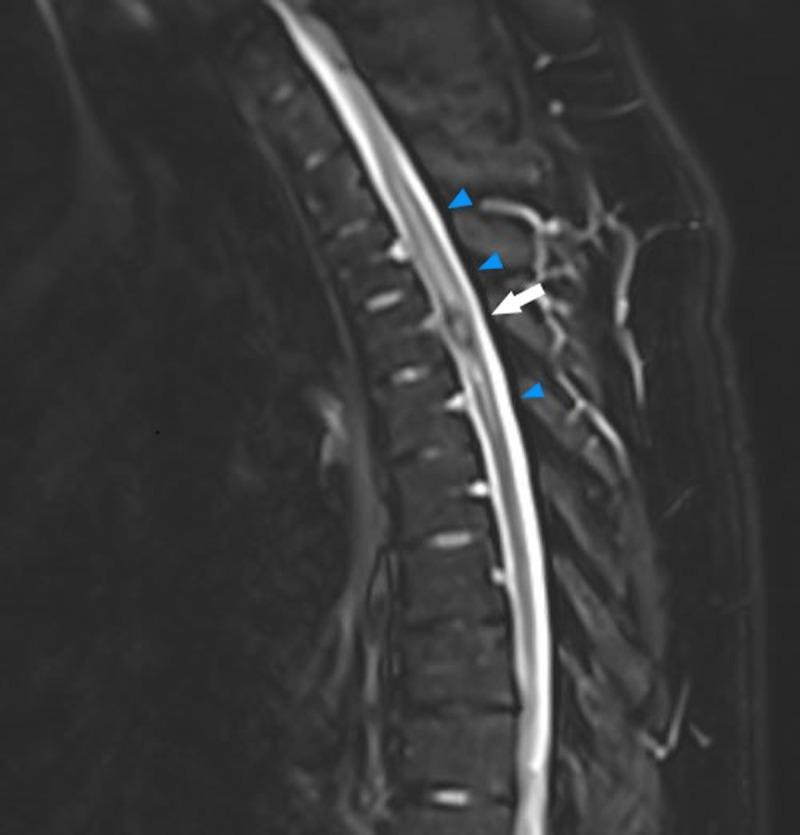


**Figure 2:**
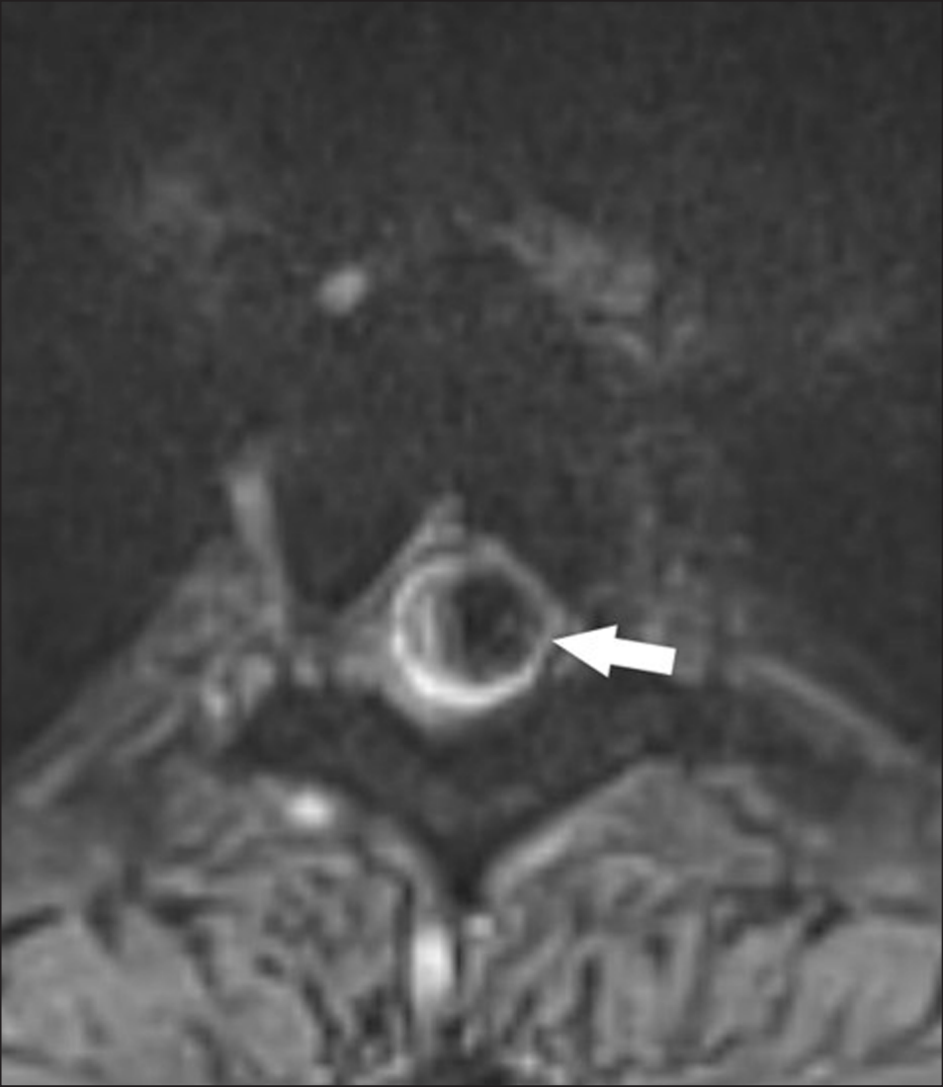


A 29-year-old male patient with a known history of familial multiple cavernous malformation syndrome (FCMS) presented with new-onset pyramidal signs during routing follow-up. Initial brain computed tomography (CT) showed multiple hyperdense cerebral lesions corresponding to known cerebral cavernous malformations (CCM), without apparent acute hemorrhage. Because of persisting symptoms, magnetic resonance imaging (MRI) of the thoracic spine was performed 10 days later. T2-weighted images (T2-WI) showed a intramedullary round hyperintense focus with a hypo-intense rim at the level of the 4th thoracic vertebral body (Th4) (Figure 1, white arrow), surrounded by hyperintense signal extending from Th2 to Th5 and medullary swelling (Figure 1, blue arrows). Intramedullary susceptibility artifacts (blooming artifacts) were encountered on axial T2*-WI (Figure 2, white arrow). In this clinical setting, findings are suggestive of a spinal cavernous malformation (SCM).

**Figure 3:**
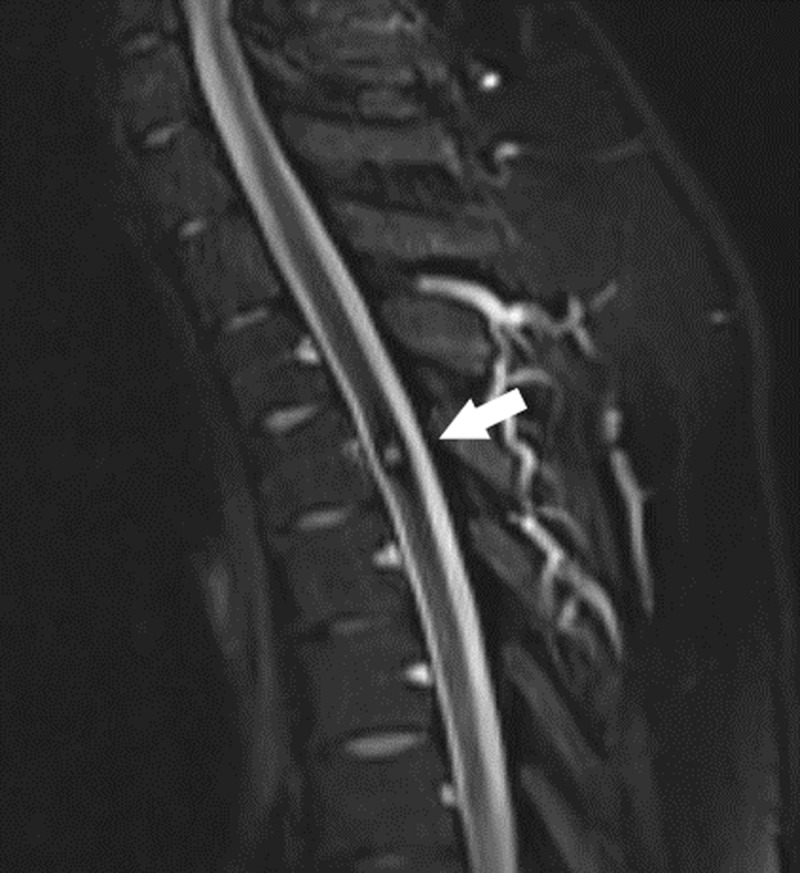


Patient was treated conservatively and recovered partially. Follow-up MR examinations after 4 and 10 months showed regression of the intramedullary edema with persistence of a central T2-hyperintense focus and blooming artifact on T2*-WI (Figure 3, white arrow).

## Comments

Cerebral and spinal cavernous malformations represent clusters of enlarged capillaries in a ‘mulberry-like’ formation without intervening brain or medullary tissue, often surrounded by hemosiderin due to recurrent microhemorrhages. These malformations can either be sporadic or inherited. FCMS is more likely when multiple cavernomas are present. Different causative genetic mutations have been identified.

SCMs are rare and make up 5% of all spinal lesions. Clinical presentation can be variable with acute or insidious neurological deficits, and SCMs are associated with significant morbidity. Most lesions are located at the level of the thoracic spine, as is the case in our patient [1].

Unenhanced CT may show larger CCM as spontaneous hyperdense foci, but smaller lesions are often overlooked. MRI is the modality of choice for evaluating disease extension and associated hemorrhages. T2*-WI and susceptibility weighted imaging are superior compared to T2-WI for detecting blood breakdown products and one of these sequences should always be performed. Cavernous malformations are radiologically subdivided into 4 categories (Zabramski calssification). Typical (type 2) lesions have a mixed T2-signal centrally with a hypo-intense hemosiderin rim. Bi-directional T2- and T1-hypo-intense extension in the spinal cord representing intramedullary hemorrhage is often seen in SCM. Unlike in CCM, popcorn-like appearance is uncommon. Appearance on T1-WI is variable [1].

Most patients are treated conservatively but surgical resection may be necessary in cases with severe or recurrent symptoms. Patients with FCMS may benefit from follow-up using brain and spinal MRI although no widely used guidelines exist.
